# Supplementary material for: Phenazine-1-carboxylic Acid Produced by Pseudomonas chlororaphis YL-1 Is Effective against Acidovorax citrulli
Source: Microorganisms. 2021 Sep 23;9(10):2012. doi: 10.3390/microorganisms9102012 (PMC8541086; doi:10.3390/microorganisms9102012)
Supplement: Supplementary file 1 [file microorganisms-09-02012-s001.zip › microorganisms-1329464-supplementary.pdf]

Supplementary Materials

Phenazine-1-carboxylic acid produced by *Pseudomonas chlororaphis* YL-1 is effective against *Acidovorax citrulli*

Youzhou Liu[a,c], Yaqiu Zhou[a], Junqing Qiao[a], Wenjie Yu[b], Xiayan Pan[a], Tingting Zhang[b], Yongfeng Liu[a,*] Shi-En Lu[c*],

[a] Institute of Plant Protection, Jiangsu Academy of Agricultural Sciences, Nanjing 210014

[b] College of Plant Protection, Nanjing Agricultural University, Nanjing 210095

[c] Department of Biochemistry, Molecular Biology, Entomology and Plant Pathology, Mississippi State University, Mississippi State, USA, 39759

*Correspondence to:

Shi-En Lu, Department of Biochemistry, Molecular Biology, Entomology and Plant Pathology, Mississippi State University, Mississippi State, MS 39762; Tel: +16623253511; fax: +16623258955.

Email: sl332@msstate.edu

Or Yongfeng Liu, Institute of Plant Protection, Jiangsu Academy of Agricultural Sciences, No.50 Zhongling Street, Nanjing 210014, China. Tel: +8602584391002.

Email: liuyf@jaas.ac.cn

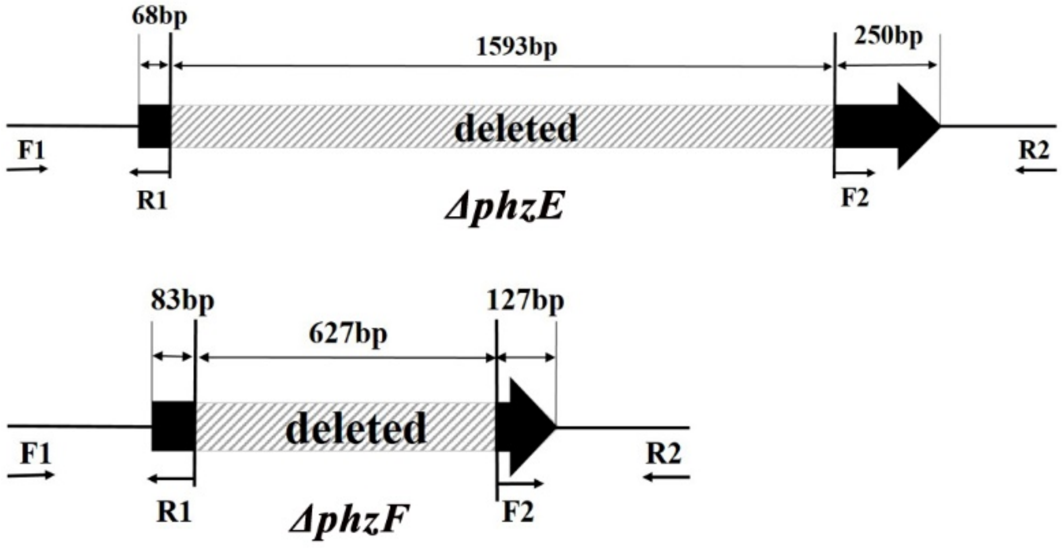

Figure S1. Two deletion mutants *ΔphzE* and *ΔphzF* of *Pseudomonas chlororaphis* YL-1. First, the upstream and downstream homolog fragments of *phzE* in wild-type strain YL-1 were cloned by using two pairs of primers (*phzE*-F1, *phzE*-R1, *phzE*-F2 and *phzE*-R2). Then, a *sacB*-containing suicide-vector, pEX18Gm was used to creating three recombinant plasmids, pEX18-*phzE*. The recombinant plasmids were first transformed into *E.coli* S17-1, then transformed into wild-type strain YL-1 via an optimized bacterial conjugal approach. Via a double-crossover homologous recombinant approach, the mutants were generated. Gene replacements were verified by PCR amplifications using specific primers and DNA sequencing. The mutant *ΔphzF* was generated as described above.

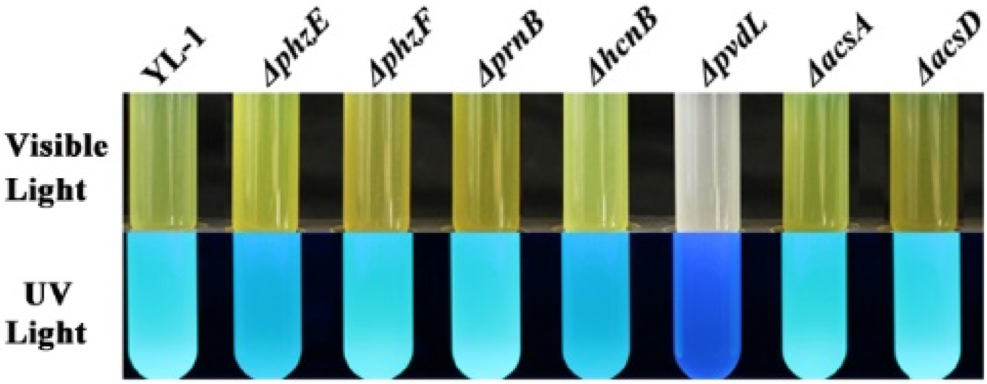

Figure S2. Comparison of the pyoverdine production between *P. chlororaphis* YL-1 and mutants cultured in liquid SM. *P. chlororaphis* YL-1 and mutants were cultured in liquid SM medium, respectively, at 28°C and 200 rpm for 24 h. All liquid cultures were observed under visible light and ultraviolet light.

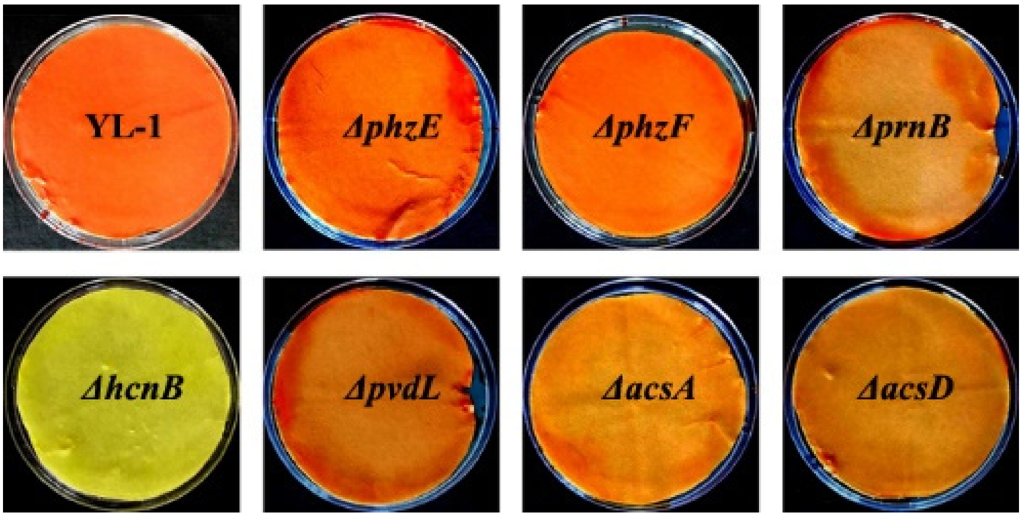

Figure S3. Comparison of the production of hydrogen cyanide between *P. chlororaphis* YL-1 and mutants cultured in King's B medium containing glycine (4.5 g/L). A sterilized filter paper saturated with 1% solution of picric acid and 2% sodium carbonate was placed in the upper lid of a petri dish. The petri dish was then sealed with parafilm and incubated at 30°C for 4 days. A change in color of the filter paper from yellow to reddish brown as an index of cyanogenic activity was recorded.

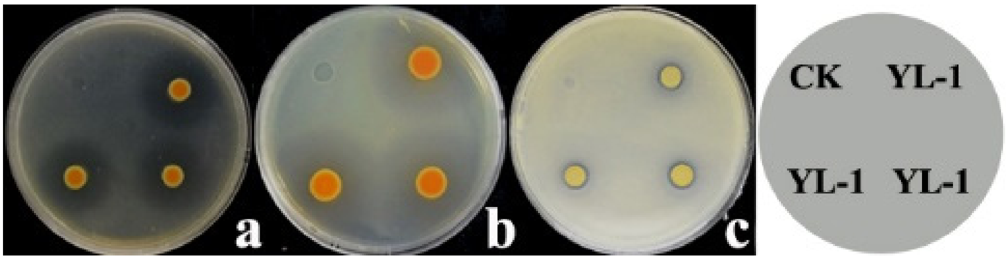

Figure S4. Antibacterial activity of *P. chlororaphis* YL-1 on LB plates. All strains precultured in liquid LB medium at 28°C and 200 rpm for 24 h were resuspended in distilled liquid LB medium to the desired cell density of 10^8 CFU/mL. After spraying the indicator bacteria onto the surface of LB plates for 1 s, 5 μL cells of strain YL-1 were added on the LB plates, 1 cm from the edge of the plate in a cross shape, and incubated at 28°C for 48 h. Distilled LB medium was used as a control. Each treatment was replicated three times, and each experiment was repeated thrice independently.

a: *A. citrulli* strain XJX12, b: *Xoo* strain PXO99, c: *Xooc* strain RS11.

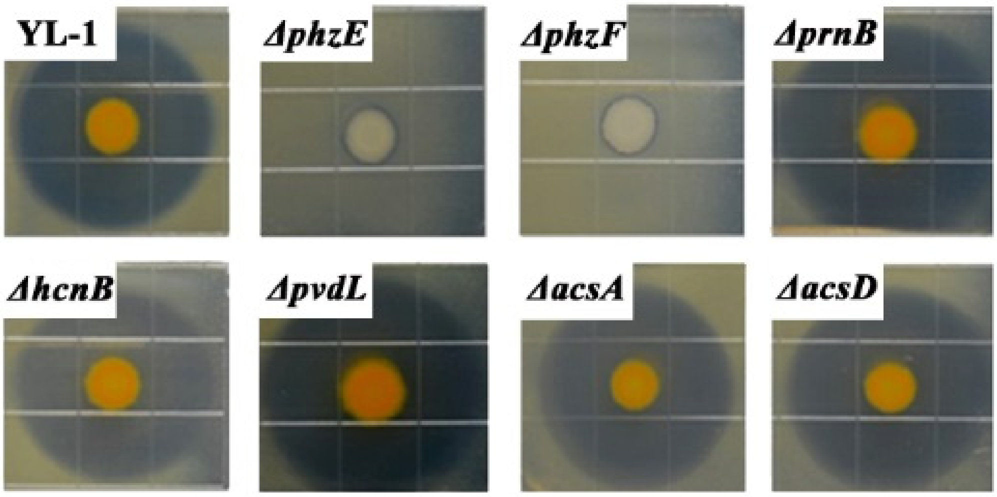

Figure S5. Anti-*Xoo* activity of *P. chlororaphis* YL-1 and its seven mutants on LB plates. All strains precultured in liquid LB medium at 28°C and 200 rpm for 24 h were resuspended in distilled liquid LB medium to the desired cell density of 10^8 CFU/mL. After spraying the suspension of *Xoo* (10^8 CFU/mL) onto the surface of LB plates for 1 s, 5 μL cells of strain YL-1 or mutants was

injected into the central hole (5 mm) of LB plates and incubated at 28°C for 48 h. Distilled LB medium was used as a control. The inhibitory zone diameters of strain YL-1 and mutants were measured when *Xoo* covered the entire control plates. Each treatment was replicated three times, and each experiment was repeated thrice independently.

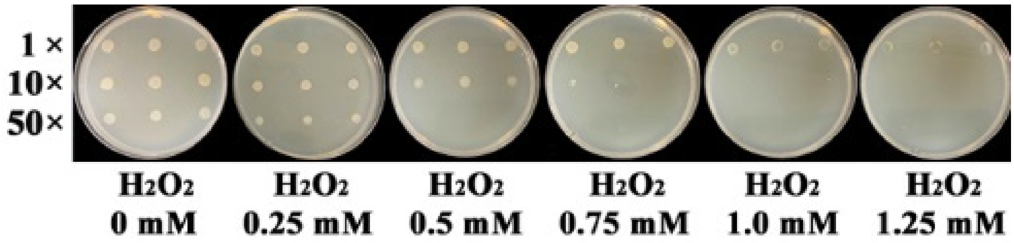

Figure S6. H_2O_2 sensitivity of *A. citrulli* on LB plates. The one-, ten-, fiftyfold dilutions (1×, 10×, and 50×) of the bacterial suspension were spotted on to LB plates, which contained different concentrations of H_2O_2 at 0, 0.25, 0.5, 0.75, 1.0, and 1.25 mM. Each treatment was replicated three times, and each experiment was repeated thrice independently.

Table S1. Strains, plasmids and primers used in this study

Items	Relevant characteristics*	Source
Pseudomonas chlororaphis		
YL-1	Wild-type strain, Amp^r	Liu (2015) [24]
ΔpvdL	*pvdL* in-frame deletion mutant of strain YL-1, Amp^r	Liu (2021) [33]
ΔphzE	*phzE* in-frame deletion mutant of strain YL-1, Amp^r	This study
ΔphzF	*phzF* in-frame deletion mutant of strain YL-1, Amp^r	This study
ΔprnB	*prnB* in-frame deletion mutant of strain YL-1, Amp^r	This study
ΔhcnB	*hcnB* in-frame deletion mutant of strain YL-1, Amp^r	This study
ΔacsA	*acsA* in-frame deletion mutant of strain YL-1, Amp^r	This study
ΔacsD	*acsD* in-frame deletion mutant of strain YL-1, Amp^r	This study
Escherichia coli		

DH5α	F^-, ϕ80*lacZ*ΔM15, Δ (*lacZYA-argF*) *U169, deoR, recA1, endA1, hsdR17* (r_k^-,m_k^+), *phoA, supE44*, $λ^-$, *thi-1, gyrA96*	Sangon Corp. collection
S17-1λpir	*(F^-)RP4-2-Tc::Mu aphA::Tn7 recA λpir lysogen*, Sm^r, Tp^r	Simon(1983) [29]
Phytopathogenic bacteria		
Acidovorax citrulli strain XJX12	Wild-type strain	Liu (2019) [37]
Xanthomonas oryzae pv. *oryzae* strain PXO99	Wild-type strain	Liu (2021) [33]
Xanthomonas oryzae pv. *oryzicola* strain RS11	Wild-type strain	Liu (2021) [33]
**Plasmids**		
pEX18Gm	Suicide plasmid with a *sacB* gene, Gm^r	Hoang (1998) [28]
pEX18-*pvdL*	pEX18GM with two flanking fragments of *pvdL*	Liu (2021) [33]
pEX18-*phzE*	pEX18GM with two flanking fragments of *phzE*	This study
pEX18-*phzF*	pEX18GM with two flanking fragments of *phzF*	This study
pEX18-*prnB*	pEX18GM with two flanking fragments of *prnB*	This study
pEX18-*hcnB*	pEX18GM with two flanking fragments of *hcnB*	This study
pEX18-*acsA*	pEX18GM with two flanking fragments of *acsA*	This study
pEX18-*acsD*	pEX18GM with two flanking fragments of *acsD*	This study
**Primers****		
pvdL-F1	5'-CG**GAATTC** GGAAGAAAATCGGCACCACG (EcoRI) -3'	Liu (2021) [33]
pvdL-R1	5'-CGG**GGTACC**GAGGAACTGGCGCAGTACAT (KpnI) -3'	Liu (2021) [33]
pvdL-F2	5'-CGG**GGTACC** CTCTGATCCTGCTCTTCGGC (KpnI) -3'	Liu (2021) [33]
pvdL-R2	5'-GC**TCTAGA** GCCTGCTTGCGATAGTGGTC (XbaI) -3'	Liu (2021) [33]
phzE-F1	5'-CGG**GGTACC**TGACCCCCAAGCCCAGTGAC (KpnI) -3'	This study
phzE-R1	5'-CCC**AAGCTT**TACAACAGGGCAAACGGCTC (HindIII) -3'	This study
phzE-F2	5'-CCC**AAGCTT**TTTCTACAACACCTTCGCCG (HindIII) -3'	This study
phzE-R2	5'-CCG**GAATTC**TCATCGGCGTCAAAGAACAC (EcoRI) -3'	This study
phzF-F1	5'-CGG**GGTACC**TCCAACAGCCGAAAATCAAC (KpnI) -3'	This study

phzF-R1	5'-CCC**AAGCTT**TCATCGGCGTCAAAGAACAC (HindIII) -3'	This study
phzF-F2	5'-CCC**AAGCTT**TCAGGGCGTGGAAATCGGCC (HindIII) -3'	This study
phzF-R2	5'-CCG**GAATTC** ATCTGCTGGCTGGTTTCGCG (EcoRI) -3'	This study
prnB-F1	5'-GG**GGTACC**GACGCATTCAACGCCGAGAT (KpnI) -3'	This study
prnB-R1	5'-CG**GGATCC**CACCCGGTCCAAGGTGCGTT (BamHI) -3'	This study
prnB-F2	5'-CG**GGATCC**CCGTGCTCGACGAATCCTGA (BamHI) -3'	This study
prnB-R2	5'-GC**TCTAGA**AACTCCTTCGGGTCGTGCTC (XbaI) -3'	This study
hcnB-F1	5'-GG**GGTACC**TGTTTTCCAGTTAGGGCAGG (KpnI) -3'	This study
hcnB-R1	5'-CCC**AAGCTT**ATCAGTGGATTCAGGCTCAT (HindIII) -3'	This study
hcnB-F2	5'-CCC**AAGCTT**GATTCCCTTTTCCGCTTTCC (HindIII) -3'	This study
hcnB-R2	5'-CG**GAATTC**CGGTCCTCGTCGTCGTAGAT (EcoRI) -3'	This study
acsA-F1	5'-GG**GGTACC**TCTTTGCTGGTGAGTTTGAT (KpnI) -3'	This study
acsA-R1	5'-CCC**AAGCTT**GCAAGGGCGAAGTGGTCAAC (HindIII) -3'	This study
acsA-F2	5'-CCC**AAGCTT**AAAGAAGTCTTCGGCCAGCA (HindIII) -3'	This study
acsA-R2	5'-CG**GAATTC**GACCTCAACCCCAAGACCCT (EcoRI) -3'	This study
acsD-F1	5'-GG**GGTACC**ACACATAGGGCTTGCGGGTC (KpnI) -3'	This study
acsD-R1	5'-CCC**AAGCTT**CGTTATGGCTGAGTTGTCCC (HindIII) -3'	This study
acsD-F2	5'-CCC**AAGCTT**ATCTTCGATAAAACATGCTT (HindIII) -3'	This study
acsD-R2	5'-CG**GAATTC**TGGCTCCAGCAGCACCATGCT (EcoRI) -3'	This study
PhzE FP	5'- CAG**GAATTC**TGCTCAAGGACTTCTGGGGC(EcoRI) -3'	This study
PhzE RP	5'- CAC**AAGCTT**CAGCTCAAAGGCGATGGTGC(HindIII) -3'	This study
RT-*oxyR*-F	5'-CGTCGCTCGAAACCATCAAGCAC-3'	This study
RT-*oxyR*-R	5'-CTGCCGTCGTCCTCCTCGAT-3'	This study
RT-*soxR*-F	5'-AACTCGACGAACGCATCGCCAC-3'	This study

RT-*soxR*-R	5'-CAACGAGAGACAGCCGCAACCG -3'	This study
RT-*katB*-F	5'-GATGGTCCTGAACAAGAACG-3'	This study
RT-*katB*-R	5'-TCCGAATACGAGAACACGCG-3'	This study
RT-*katE*-F	5'-TGGATGAAGAACACGGGGAT-3'	This study
RT-*katE*-R	5'-TGTTCTGCCGCTTCTCCACG-3'	This study
RT-*katG*-F	5'-AGGTGCCGAAGGAAGAACTG-3'	This study
RT-*katG*-R	5'-AGTTGCTCGGGCTGGTTGAC-3'	This study
RT-*ahpC*-F	5'-GTAGTTGTCGGCGGCGTCTT-3'	This study
RT-*ahpC*-R	5'-CAAGTGGTCCGTGCTGATCT-3'	This study
RT-*ahpF*-F	5'-ATCCTCAATCCCCGCATCCG-3'	This study
RT-*ahpF*-R	5'-GCCGTTGAGGAAGACCATGG-3'	This study
RT-*trxA*-F	5'-TACTGGTGGACTACTGGGCC-3'	This study
RT-*trxA*-R	5'-TGAGCTTGCCCTGGTAGGAG-3'	This study
RT-*sodA*-F	5'-GTCAACAATCTGAACGCCGC-3'	This study
RT-*sodA*-R	5'-AACGCATCGAAACCGCCCAG-3'	This study
RT-*sodB*-F	5'-CCGCACTACAGCCAGGAAAC-3'	This study
RT-*sodB*-R	5'-TGGGCGGCGTTGTTGTAGAT-3'	This study
RT-*sodC*-F	5'-GCTTCCACATCCACGCCAAT-3'	This study
RT-*sodC*-R	5'-TGATGTTGGGCAGTTCGCCG-3'	This study
XJ-RT-F	5'-AGCCATTTCCCCGTCCAGAC-3'	Liu (2019) [37]
XJ-RT--R	5'-CGGGGCTCGAAGCTCCCGTAG-3'	Liu (2019) [37]

*Amp^{R} , Gm^{R}, resistance to ampicillin and gentamicin, respectively.

** Restriction enzyme digestion sites are in bold.
